# Extramedullary Relapse in a CML Patient after Allogeneic Stem Cell Transplantation

**DOI:** 10.1155/2017/6350267

**Published:** 2017-03-21

**Authors:** Asu Fergun Yilmaz, Nur Soyer, Nazan Ozsan, Seckin Cagirgan, Ajda Gunes, Melda Comert, Fahri Sahin, Guray Saydam, Nur Selvi Gunel, Filiz Vural

**Affiliations:** ^1^Hematology Department, Ataturk Training and Research Hospital, İzmir Katip Celebi University Hospital, İzmir, Turkey; ^2^Hematology Department, Ege University Hospital, İzmir, Turkey; ^3^Pathology Department, Ege University Hospital, İzmir, Turkey; ^4^Hematology Department, Medical Park Hospital, İzmir, Turkey; ^5^Hematology Department, Sivas Numune Hospital, Sivas, Turkey; ^6^Hematology Department, Inonu University Hospital, Malatya, Turkey; ^7^Medical Biology Department, Ege University Hospital, İzmir, Turkey

## Abstract

Myeloid or granulocytic sarcoma (GS) is a tumoral lesion consisting of immature granulocytic cells. It is a rare entity during the course of CML patients especially after allogeneic stem cell transplantation (SCT). Relapse without bone marrow involvement is much rarer. We report a case of CML patient who relapsed with isolated granulocytic sarcoma after allogeneic SCT during cytogenetic and molecular remission. 28-year-old male was diagnosed as CML and allogeneic SCT was performed because of refractory disease to tyrosine kinase inhibitors. Complete cytogenetic and molecular response was achieved after allogeneic SCT followed by dasatinib treatment. Approximately 5 years after the transplantation, very rapidly progressive lesion was documented and diagnosed as GS although he was at molecular and cytogenetic remission. The patient died during chemotherapy due to sepsis. GS relapse after allogeneic SCT is a very rare type of relapse in CML patients with molecular and cytogenetic remission. Since it is a very aggressive disease with a poor prognosis, combined chemoradiotherapies with other possible options like DLI or second allogeneic SCT should be considered as soon as the diagnosis is confirmed.

## 1. Introduction

Myeloid or granulocytic sarcoma (GS) is a tumoral lesion consisting of immature granulocytic cells. They may arise de novo or may accompany mostly acute myeloid leukemia. Less frequently associations with myeloproliferative diseases, myelodysplastic syndrome, and chronic myeloid leukemia (CML) were documented in the literature [1–3]. Most of the GS cases in CML patients were diagnosed in patients without cytogenetic remission or patients in blastic phase. GS is a rare entity during the course of CML patients especially after allogeneic stem cell transplantation (SCT) [4–6]. Relapse without bone marrow involvement is much rarer. Although GS relapse of CML is accepted as a highly refractory disease and a combination chemoradiotherapies with or without second allogeneic transplantation is usually preferred, little is known about predisposing factors, natural history, and response to treatment of these patients as compared to marrow relapse.

We report a case of CML patient who relapsed with isolated granulocytic sarcoma after allogeneic SCT when he was at cytogenetic and molecular remission.

## 2. Case

**Figure 1:**
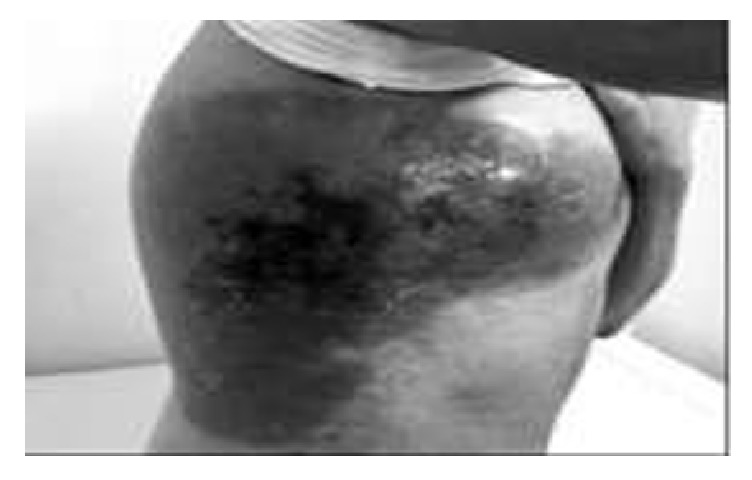


**Figure 2:**
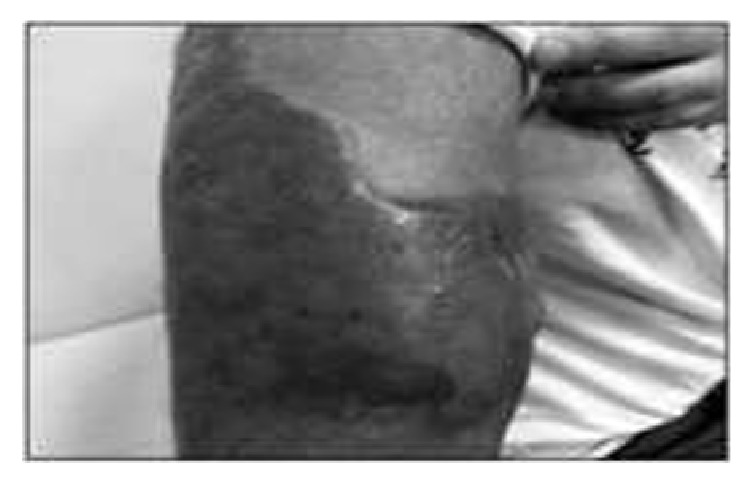


A 28-year-old male was diagnosed as CML. The cytogenetic analysis was 46 XY,+(9,22)(q34,q22) Ph in all of the 13 metaphases. He was first treated with hydroxyurea and interferon. After the tyrosine kinases were available, the treatment was changed with imatinib mesilate. He used imatinib mesilate irregularly and he was diagnosed as accelerated phase CML. The dose of the drug increased to 800 mg/day but we could not achieve response. Allogeneic SCT was performed from his full-matched male donor during the accelerated phase. After the transplantation, although the complete cytogenetic response was achieved, there was no molecular response. Under these circumstances, dasatinib 100 mg/day was started and he was followed up in complete cytogenetic and molecular responses. Approximately 5 years after the transplantation, a small lesion with a dimension of 1 × 2 cm was documented. Under the antibiotic treatment the lesion progressed rapidly to a huge, painful lesion with a diameter of 15 × 20 cm starting from his shoulder and spreading to his back and under his axillary region (Figures 1 and 2).

**Figure 3:**
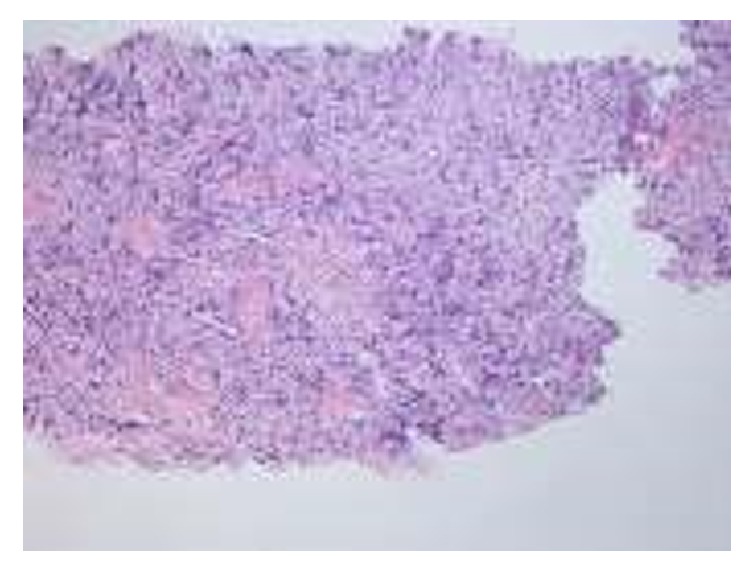
Hematoxylin and eosin specimen demonstrating infiltrative cells with high mitotic and apoptotic indices.

**Figure 4:**
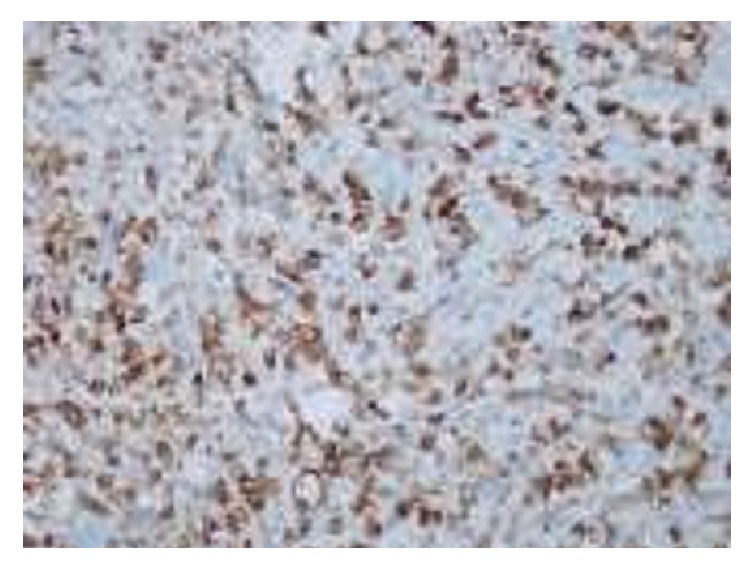
The infiltrative cells are CD34 and CD68 positive.

The biopsy of the lesion was reported as granulocytic sarcoma. The infiltrative cells had a high mitotic and apoptotic index. The cells had a Kİ67 proliferation index of 80%. The cells were CD34 and CD68 positive (Figures 3 and 4). The granulocytic sarcoma was used for bcr/abl detection. With total RNA isolation from paraffin tissue material of the case, the c DNA synthesis was carried out. Then t(9,22) translocation was revealed by q RT-PCR and quantified. The IS was 9.1552.

**Figure 5:**
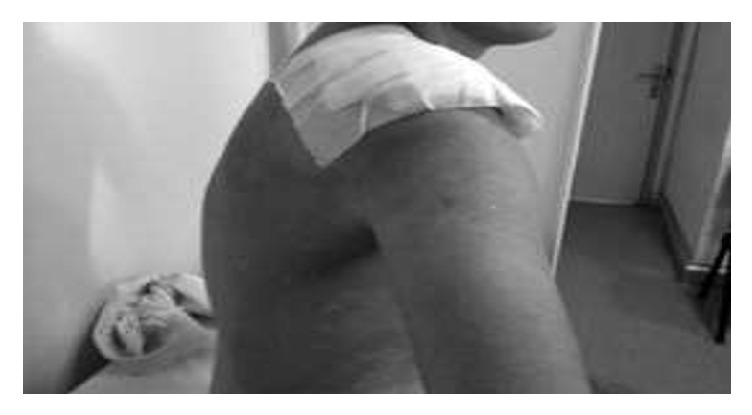
The regression after combination chemotherapy.

The physical examination and laboratory tests were normal. The bone marrow biopsy and aspiration was normocellular without any sign of CML. He was in molecular and cytogenetic remission. The cytogenetic analysis was 46 XY and molecular tests reveal no bcr/abl. Peripheral blood was used for bcr/abl PCR. The quantification of t(9,22) was performed via real time q RT-PCR. LightCycler-t(9;22) quantification kit ensures a quantitative measurement of BCR-ABL fusion transcripts, resulting from both M-bcr and m-bcr breakpoints. First, radiotherapy and subsequently chemotherapy regimen (3 + 7; 3 days of idarubicin and 7 days of ARA-c) was administered. More than 90% regression was achieved after the combination treatment (Figure 5).

After few weeks, the lesion progressed. He was discussed in Allogeneic Transplantation Council and second transplantation from the same donor was planned after the control of the lesion. FLAG-ida was administered with palliative radiotherapy. But, during the chemotherapy, he died because of sepsis and uncontrolled infection.

## 3. Discussion

GS, tumor composed of immature blastic cells, may arise at any site of the body including body cavities, neck, skull, limb, and trunk regions [2]. In our case, the mass appearing in the trunk progressed rapidly to the upper extremity. It may occur de novo or accompany other hematologic malignancies [1]. Relapse with GS in a CML patient after allogeneic SCT is a rare entity. In a retrospective study by European Bone Marrow Transplantation Registry only 0,2% of CML/MDS patients relapsed as a GS after allogeneic SCT [6].

In our case, the cytogenetic analysis revealed 46 XY genotype. t(9,22) was not detected by cytogenetic analysis or FISH. Although the marrow was in complete cytogenetic and molecular remission, how a GS could progress was obscure. This may be explained by inefficacy of immune surveillance function or graft versus leukemia effect outside the bone marrow cavity. This phenomenon might be due to lack of a suitable milieu of cytokines or other molecules for the effector cells to function maximally in these tissues [7], but it was difficult to confirm that this was due to lack of enough graft versus leukemia effect especially in tissues other than bone marrow.

In our case, the relapse was documented five years after transplant during the molecular remission. After the transplantation the molecular remission was achieved with dasatinib. In a study with CML patients treated with allogeneic stem cell transplantation, it was concluded that molecular studies during the first 5 years after transplant could help to predict long-term leukemia-free survival. The patients with negative results during first 5 years after transplant period were at low risk of relapse [8]. In our case negative results were achieved after initiation of dasatinib treatment and relapse with isolated GS occurred under dasatinib and molecular remission.

In the literature CML relapse with GS after allogeneic stem cell transplantation was a rare entity [6] so effective treatment option for these patients was not well documented. Combined aggressive chemoradiotherapy and second allogeneic stem cell transplantation in favorable patients was mostly preferred. Under these circumstances, the survival rates were still very short. The survival was reported as few as several months and very few people could survive more than 2 years [9–12].

Since the survival was very poor, all possible treatment options like second transplantation and donor lymphocyte infusions should be tried. But although DLI was an effective treatment for marrow relapse, its effect on extramedullary disease without marrow involvement was not well documented. In our case, the patient relapsed just after the chemotherapies which indicated a highly refractory disease. He could survive only for 8 months after the relapse.

In conclusion, GS relapse in CML patients with allogeneic SCT is a very aggressive disease with a poor prognosis. So combined chemoradiotherapies with other possible options like DLI or second allogeneic SCT should be considered in these patients as soon as the diagnosis is confirmed. Although it is a rare disease, prospective randomized studies are needed to define the optimal treatment for these patients.
